# Willingness to receive text message medication reminders among patients on antiretroviral treatment in North West Ethiopia: A cross-sectional study

**DOI:** 10.1186/s12911-015-0193-z

**Published:** 2015-08-13

**Authors:** Mihiretu Kebede, Atinkut Zeleke, Mulusew Asemahagn, Fleur Fritz

**Affiliations:** Department of Health Informatics, Institute of Public Health, College of Medicine and Health Sciences, University of Gondar, Gondar, Ethiopia; Institute of Public Health, College of Medicine and Health Sciences, University of Gondar, Gondar, Ethiopia; Bahirdar University, Bahirdar, Ethiopia; Institute of Medical Informatics, University of Muenster, Münster, Germany

**Keywords:** Text message, Medication reminders, ART, Cellphone, Willingness, mHealth

## Abstract

**Background:**

Non-adherence to Antiretroviral Treatment (ART) is strongly associated with virologic rebound and drug resistance. Studies have shown that the most frequently mentioned reason for missing ART doses is the forgetfulness of patients to take their medications on time. Therefore using communication devices as reminder tools, for example alarms, pagers, text messages and telephone calls could improve adherence to ART. The aim of this study is to measure access to cellphones, willingness to receive text message medication reminders and to identify associated factors of ART patients at the University of Gondar Hospital, in North West Ethiopia.

**Methods:**

An institution based cross sectional quantitative study was conducted among 423 patients on ART during April 2014. Data were collected using structured interviewer-administered questionnaires. Data entry and analysis were done using Epi-Info version 7 and SPSS version 20 respectively. Descriptive statistics and multivariable logistic regression analysis were used to describe the characteristic of the sample and identify factors associated with the willingness to receive text message medication reminders.

**Results:**

A total of 415 (98 % response rate) respondents participated in the interview. The majority of respondents 316 (76.1 %) owned a cellphone, and 161(50.9 %) were willing to receive text message medication reminders. Positively associated factors to the willingness were the following: Younger age group (AOR = 5.18, 95 % CI: [1.69, 15.94]), having secondary or higher education (AOR = 4.61, 95 % CI: [1.33, 16.01]), using internet (AOR = 3.94, 95 % CI: [1.67, 9.31]), not disclosing HIV status to anyone other than HCP (Health Care Provider) (AOR = 3.03, 95 % CI: [1.20, 7.61]), availability of radio in dwelling (AOR = 2.74 95 % CI: [1.27, 5.88]), not answering unknown calls (AOR = 2.67, 95 % CI: [1.34, 5.32]), use of cellphone alarm as medication reminder (AOR = 2.22, 95%CI [1.09, 4.52]), and forgetting to take medications (AOR = 2.13, 95 % CI: [1.14, 3.96]).

**Conclusions:**

A high proportion of respondents have a cell phone and are willing to use it as medication reminders. Age, educational status and using internet were the main factors that are significantly associated with the willingness of patients to receive text message medication reminders.

**Electronic supplementary material:**

The online version of this article (doi:10.1186/s12911-015-0193-z) contains supplementary material, which is available to authorized users.

## Background

Since the introduction of Antiretroviral Treatment (ART) in developed nations during the mid-1990s, access to ART has become more widely available. This combination of drugs has fundamentally transformed the lives of People Living with HIV/AIDS (PLWHA) and the Human Immunodeficiency Virus (HIV) infection has changed from a serious and deadly illness to a more easily manageable disease [1]. Once the treatment has started the patient needs to strictly adhere (optimal adherence, 95 %) to it throughout his/her entire lifetime to maintain the functionality of the immune system of the individual and to control the emergence of drug resistant strains [1, 2].

According to the World Health Organization (WHO), more than 50 % of all medicines are prescribed, dispensed or sold inappropriately, and half of all patients fail to take medicines correctly [2].

Non-adherence to ART and forgetting to take medications are strongly associated with virologic rebound and clinically significant resistance. A cumulative adherence of 70–89 % was strongly associated with viral rebound and clinically significant resistance, when compared with cumulative adherence of 90 % [3]. Patients who used medication reminders were less likely to develop resistance [3]. The prevalence of non-adherence to ART in Ethiopia was revealed to be high (17.3 %) in a study done in Felegehiwot Hospital and University of Gondar Hospital [4].

Missing healthcare appointments could be a consequence of inefficient healthcare delivery, with ample expenses for the health system, leading to delays in diagnosis and treatment. Forgetfulness is the main reason for missing healthcare appointments [5, 6] and ART doses [7]. Hence, communication devices which act as a reminding tool e.g. alarms [8], pagers, and telephones could supplement adherence support strategies [1, 9]. Reminders in the form of text messages, alarm tones, calls to landlines or cellphones may help patients meet their healthcare appointments and remember to take their medications accordingly [5, 8, 10, 11].

Previous studies conducted in Africa and elsewhere have demonstrated that text messaging or cellphone call medication reminders significantly improve the adherence of patients on ART [11–13].

Cellphones are the most ubiquitous types of equipment in the world and nearly one of every two citizens of the planet possesses a cellphone [14]. Many African countries have reached more than 30 % mobile cellular telephone subscription rates, markedly lower than the world average of 78 % [15]. Sub Saharan Africa has registered the highest cellphone uptake rate in the world and the mobile network coverage rate is rapidly expanding [16]. In Ethiopia, mobile subscribers have reached 17 % of the total population and the mobile network coverage is expanding [17]. In 2012, mobile network coverage of the country has reached 73 % [18].

There is a great opportunity to link the ever-growing mobile telecommunication technology with the multifaceted ART adherence support strategies. An electronic health service readiness assessment study from Ethiopia concluded that mobile-based health services are feasible for consultation, creating awareness, and diagnosis and treatment because the affordability of mobile phones by low income inhabitants and the growth of the mobile network coverage of the country is increasing [19].

However, there are also challenges. As Tamaryn C. et al. indicated: the loss of cellphone devices due to theft and/or damage, the patterns of cellphone use and privacy issues influence the willingness of patients to receive mobile phone text or call reminders [20]. In some settings, one mobile phone might be used by more than one individual and therefore cellphone interventions will need to consider issues of confidentiality and privacy [13, 21].

Although ART has dramatically improved the health of patients and reduced the morbidity and mortality of HIV patients in Ethiopia, adherence to ART is still a problem and a significant contributor to drug resistance and treatment failure. A number of strategies have been tried to enhance the multifaceted issue of treatment adherence, but very little has been done using mobile telecommunication technology.

Access to cellphone technology among patients on ART and the willingness of patients to receive text message medication reminders are still unknown. Before implementing cellphone text message based treatment adherence strategies, the willingness of patients to receive text message medication reminders needs to be investigated.

Therefore the objectives of this study are to:

- determine the access to cellphones among patients on ART,
- determine the willingness of those patients to receive text message medication reminders and
- identify the factors associated with the willingness to receive text message medication reminders

## Methods

### Study design and setting

An institution based cross sectional quantitative survey was conducted at the University of Gondar Hospital in April 2014. This hospital serves a population of more than five million. It is a tertiary level hospital in the Ethiopian three tier healthcare system, located 727 km North West of Addis Ababa. Nearly 10,000 patients are currently receiving ART in this hospital.

### Study subjects

All HIV patients whose age is > = 15 years and are taking their ART medication at the University of Gondar Hospital were the source population for this study. A systematic random sampling technique was performed to select 423 study participants. The sample size of this study was determined using the single population proportion formula (*n* = Z_(α/2)_^2^pq/∂^2^) [22, 23] with the following assumptions:

n = the required sample size

Z = the value of standard normal distribution corresponding to α/2, 1.96

p = proportion of patients who are on ART and willing to be contacted by cellphone

q = 1-p, proportion of patients who are on ART and NOT willing to be contacted by cellphone

∂ = Precision as 0.05

We could not find any study conducted to determine the access to cellphones among patients on ART, however, the general population’s access to cellphones in Ethiopia is 17 % [17]. As we also could not find any study conducted in Ethiopia to determine the willingness of patients on ART to receive text message medication reminders we assumed the proportion to be 50 %. With those numbers two sample sizes were calculated. The maximum sample size was found to be 384 using the proportion of patients who are on ART and willing to be contacted by cellphone. Taking a 10 % non-response rate into account, we calculated the final sample size to be 423.

### Study variables

According to our research objectives the primary outcome measures are: access to cellphone and willingness to receive text message ART medication reminders.

**Fig. 1:**
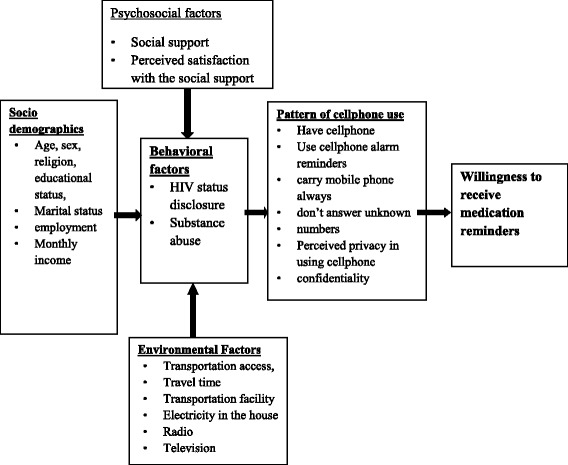
Conceptual framework (adopted from [20, 24–29])

By reviewing the existing literatures on mobile health [20, 24–29], the following independent variables were used to develop the conceptual framework for the questionnaire, which is also presented in Fig. 1:

- Socio-demographics: Age, Sex, Marital status, Educational status, Employment status
- Behavioral factors: HIV status disclosure, Substance abuse, Taking medication in front of others, Use of medication reminder mechanisms
- Psychosocial factors: Social support, Perceived satisfaction with the social support, Self-esteem, Perceived satisfaction in being valued or esteemed by others, Perceived doubts about HIV/ART and healthcare provider, Perceived treatment benefit, Perceived self confidence in taking the medication and the doses as prescribed by the clinician
- Environmental factors: Transportation access, Travel time, Transportation facility, Electricity in the house, Access to radio, Access to television
- Patient provider relationship: Level of relationship with clinician, Perceived satisfaction of patient provider relationship, Frequency of health visit, Perceived satisfaction with progress after starting ART, Missing appointment, forgetting to take medication.
- Pattern of cellphone use: Use of cellphone alarm reminders, Preferred way of communication in cellphone, Carry cellphone always, Lock cellphone with password, Do not answer unknown numbers, Perceived privacy in using cellphone, Switch off cellphone during day, Put cellphone in a place where others could use and access, Share cellphone with others, Ability to send/receive/read text messaging, Perceived text message confidentiality, Use of internet with cellphone

### Methods for data acquisition and analysis

Data were collected using structured interviewer-administered questionnaires (Additional file 1) which included the above mentioned variables and their willingness to receive text message ART medication reminders. The questionnaire was primarily prepared in English and translated in to local language, Amharic and back again to English by language experts to check its consistency. A pretest was conducted at Felegehiwot Referral Hospital with 43 participants (10 % of the total sample size). The questionnaire was modified according to the feedback from the pretest. A one day training on the objective and relevance of the study, confidentiality of data, respondents’ rights, informed consent and data collection techniques was given to three nurses who were recruited to interview the study participants. Ethical clearance and support letters were obtained from the University of Gondar ethical approval committee and the University of Gondar Hospital. Verbal consent was requested from all respondents for their willingness to participate in the study after explaining the objective of the study and data confidentiality.

The investigators conducted daily supportive supervision of the data collection process. Data from the respondents were checked for completeness and consistency before being entered into the computer for cleaning and analysis.

Data were entered using Epi-Info version 7 and transferred to SPSS version 20. Descriptive statistics were performed to describe the study population. Binary logistic regression was computed to analyze the effect of each study variable on the outcome variable. Variables significantly associated with the outcome variable (*p* < 0.05) in the bivariate analysis were subjected in a multivariable logistic regression analysis to evaluate the consistency of the effect after adjusting other variables. The strength of associations was described using Odds Ratio (OR) and a 95 % confidence interval (CI). In the bivariate and multivariable regression analysis, the total number of patients who owned cellphone (316) were included.

## Results and discussions

A total of 423 study subjects were approached for the interview, 415 (response rate 98 %) of them gave their consent and responded to the questions. The socio-demographic characteristics can be found in Table 1. The majority of respondents, 275 (66.3 %) were females and the mean age was 33.6 years (SD = 10.02). A large number of respondents, 177 (42.7 %) were married, 295 (71.1 %) had primary education, 220 (53 %) were unemployed, and 179 (43.2 %) were earning less than 25 USD per month.

**Table 1:** Socio-demographic characteristics of PLWHA at the University of Gondar Hospital, North West Ethiopia, 2014

Socio-demographic characteristics		Patients on ART (*n* = 415)		On ART and owned cellphone (*n* = 316)	
		Number	Percent	Number	Percent
Sex	Male	140	33.7	108	34.2
	Female	275	66.3	208	65.8
Age	15–30	171	41.2	124	39.2
	31–45	199	48	158	50
	>45	45	10.8	34	10.8
Marital status	Single	94	22.7	60	19
	Married	177	42.7	142	44.9
	Separated	15	3.6	13	4.1
	Divorced	78	18.8	63	19.9
	Widow/Widower	51	12.3	38	12
Educational status	No formal education	75	18.1	35	11.1
	Primary	295	71.1	238	75.3
	Secondary and above	45	10.8	43	13.6
Employment status	Unemployed	220	53	147	46.5
	Employed	195	47	169	53.5
Time since HIV diagnosis	0 to 6 months	13	3.1	9	2.8
	7 to 12 months	11	2.7	5	1.6
	>12 months	391	94.2	302	95.6
Time since ART started	0 to 6 months	26	6.3	21	6.6
	7 to 12 months	12	2.9	6	1.9
	>12 months	377	90.8	289	91.5
Income (USD per month)	Less than 25	179	43.2	114	36
	25 up to 50	115	27.7	95	30.1
	50 up to 75	26	6.3	91	28.8
	>75	95	22.9	16	5.1
Cellphone ownership	Yes	316	76.2	316	100
	No	99	23.8		

As can be seen in Table 1, 76.1 % of patients owned a cellphone. From those patients owning a cellphone, majority were females (65.8 %), and the mean (SD) age was 34.03 years (9.4). Almost half of the respondents were married 142 (44.9 %), three quarters 238 (75.3 %) had primary education, more than half 169 (53.5 %) were unemployed and more than a third 114 (36 %) were earning less than 25 USD per a month.

### Patterns of cellphone use

About three quarters (76.1 %, 95 % CI: 71.6–80.0 %) of the respondents reported having a personal cellphone. We have calculated the patterns of cellphone use percentages only from total number of patients who owned a cellphone (316). From those patients who owned a cellphone, more than 70 % (225) reported that they already use the alarm function of their cellphone as a medication reminder. For 273 (86.4 %) of patients who owned a cellphone, the preferred way of routine cellphone communication is voice call. The majority, 228 (72.2 %) of patients described that they are able to read and send text messages using their cellphone. But a large number of respondents, 163 (51.6 %) reported that their cellphone was lost, damaged or stolen in the past. Only a third of them 93 (29.4 %) lock their cellphone with a password and 169 (53.5 %) of the patients store their cellphone in a place where others can see and access it easily. More than 41 % of respondents described that they share their cellphone with others. A small number of patients, 62 (19.6 %) described that they access the internet by using their cellphone. From those patients who are using internet on their cellphone, 54 (87.1 %) reported that they are Facebook or other social network site users.

### Willingness to be contacted by ART clinic through cellphone

As summarized in Table 2, almost all respondents (95.9 % of the 316) indicated that they are willing to be contacted by the ART clinic through voice call, text messages or both. Most respondents were willing to be contacted verbally (70 %), half of them prefer text messages (50.9 %, 95 % CI: 45.3–56.3 %) and they believed that it could improve their adherence to medication. Moreover, respondents were also asked to specify what sort of service they would like to have if the ART clinic starts text message based health services. Of the total respondents owning a cellphone, 247 (78.2 %) reported they would like to have health advice or tips and 188 (59.5 %) wanted to receive text message appointment reminders.

**Table 2:** Patterns of cellphone use and willingness to be contacted through cellphone among patients on ART at University of Gondar Hospital, North West Ethiopia, 2014 (*n* = 316)

		Number	Percent
Use cellphone as medication reminder	Yes	225	71.2
	No	91	28.8
Preferred way of routine cellphone communication	Voice call	273	86.4
	Text	40	12.9
	Email	3	0.3
How often do you have your cellphone with you	Always	271	85.8
	Not always	45	14.2
Cellphone, damaged, lost, or stolen in the past	Yes	163	51.6
	No	153	48.4
Have other phone number	Yes	79	25
	No	237	75
Switch off cellphone during the day	Yes	58	18.4
	No	258	81.6
There are times or places where no calls are answered	Yes	119	37.7
	No	197	62.3
There are times, places or situations that unknown calls are answered	Yes	123	38.9
	No	193	61.1
Store cellphone where others could use and access	Yes	169	53.5
	No	147	46.5
Share cellphone with others	Yes	131	41.5
	No	185	58.5
Lock cellphone with password	Yes	93	29.4
	No	223	70.6
Read and send text messages with cellphone	Yes	228	72.2
	No	88	27.8
Likelihood of text message to be seen by others	Very Likely	69	21.8
	Likely	132	41.8
	Unlikely	25	7.9
	Very Unlikely	90	28.5
Use internet with cellphone	Yes	62	19.6
	No	254	80.4
Willingness to be contacted by cellphone(voice call, text or both)	Yes	303	95.9
	No	13	4.1
Willingness to receive text message ART medication reminders	Yes	161	50.9
	No	155	49.1
Willingness to pay for text message ART medication reminders	Yes	269	85.1
	No	47	14.9

From the total respondents who are using cell phones, (85.1 %) indicated that they are willing to pay for text message ART medication reminders based on the current tariff. Only 13 (4.1 %) patients reported that they are not willing to receive any kind of cellular phone contact from the ART service provider. Twelve of them (92 %) believed that text messages written about their medication would ruin their privacy.

### Factors associated with willingness to receive text message ART medication reminders

The bivariate analysis indicated that age, educational status, employment status, income, availability of television in dwelling, radio in dwelling, travel time, frequency of visiting ART clinic, ever miss healthcare appointment, missing medications, HIV status disclosure, substance abuse, use cellphone as medication reminder, lock cellphone with password, perceived confidentiality of text message, use internet, not answering phone calls, not answering unknown calls were significantly associated (*p* < 0.05) with the willingness of respondents to receive text message ART medication reminders. All of these variables were included in the final multivariable logistic regression model to control the effect of confounding.

The multivariable logistic regression analysis pointed out the following factors to be significantly associated with the willingness to receive text message ART medication reminders: younger age group (15–30 years; *p* = 0.004), educational status (Secondary and above; *p* = 0.016), using internet (*p* = 0.002), not disclosing HIV status (*p* = 0.019), availability of radio in dwelling (*p* = 0.01), not answering unknown calls (*p* = 0.005) , use the alarm function of cellphone as medication reminder (*p* = 0.029), and forget to take medications (*p* = 0.017).

As shown in Table 3, respondents from the age group of 15–30 years are 5.18 times more likely to be willing to receive text messages than those who are greater than 45 years of age. Respondents who had secondary and higher education are 4.61 times more likely to be willing to receive text message ART medication reminders. Respondents who use internet on their cellphone are 3.94 times more likely to be willing to receive text message medication reminders.

**Table 3:** Factors associated with the willingness to receive text message medication reminders among patients on ART at the University of Gondar Hospital, North West Ethiopia, (*n* = 316)

Factor		Willingness		Crude OR(95 % CI)	*P* value	AOR (95 % CI)	*P* value
		Yes	No				
Age	15–30	77	47	5.32(2.23, 12.73)	<0.001	5.18(1.69, 15.94)	0.004
	31–45	76	82	3.01(1.29, 7.06)	0.011		
	>45	8	26	1			
Educational status	No formal education	8	27	1			
	Primary education	123	115	3.61 (1.58, 8.27)	0.002		
	Secondary and above	30	13	7.79(2.80, 21.66)	<0.001	4.61(1.33,16.01)	0.016
Employment status	Unemployed	61	86	1			
	Employed	100	69	2.04(1.3, 3.2)	0.002		
Income (USD per month)	<51	92	113				
	> = 51	69	42	2.012(1.26, 3.24)	0.004		
TV in dwelling	Yes	141	119	2.13(1.17, 3.88)	0.013		
	No	20	36	1			
Radio in dwelling	Yes	145	108	3.94(2.12, 7.33)	<0.001	2.74(1.27, 5.88)	0.010
	No	16	47	1			
Travel time	Less than 1 h	140	115	2.32(1.29, 4.15)	0.005		
	More than 1 h	21	40	1			
Frequency of Visiting ART clinic	Every month	100	57	2.82(1.79, 4.45)	<0.001		
	> one month	61	98	1			
Ever missed healthcare appointment	Yes	77	49	1.98(1.25, 3.14)	0.003		
	No	84	106	1			
Forget to take medication	Yes	90	55	2.31(1.47, 3.63)	<0.001	2.13(1.14, 3.96)	0.017
	No	71	100	1			
HIV status disclosure	Yes	128	142	1			
	No	33	13	2.82(1.42, 5.59)	0.003	3.03(1.20, 7.61)	0.019
Substance abuse	Yes	17	6	2.93(1.12, 7.65)	0.028		
	No	144	149	1			
Use cellphone as medication reminder	Yes	125	100	1.19(1.16, 3.14)	0.011	2.22(1.09, 4.52)	0.029
	No	36	55	1			
There are times or places where no calls are answered	Yes	71	48	1.76(1.11, 2.79)	0.016		
	No	90	107	1			
There are times or places that don’t answer unknown calls	Yes	84	39	3.25(2.1, 5.23)	<0.001	2.67(1.34, 5.32)	0.005
	No	77	116	1			
Lock cellphone with pass word	Yes	60	33	2.2(1.33, 3.62)	0.002		
	No	101	122	1			
Perceived text message confidentiality	High	24	45	1			
	Low	137	110	2.34(1.34, 4.07)	0.003		
Use internet	Yes	52	10	6.92(3.36, 14.23)	<0.001	3.94(1.67, 9.31)	0.002
	No	109	145	1			

Perceived satisfaction with the clinical service, health education, access to reliable pharmacy, perceived satisfaction of the health progress after starting ART, frequency of ART clinic visits and psychosocial factors were not shown to be significantly associated with text message medication reminders. Moreover, cellphone usage privacy variables like locking cellphone with password, perceived text message confidentiality, sharing cellphone with others, storing cellphone in a place where others could see and access were not found to be significantly associated with willingness.

## Discussion

The purpose of this study was to assess the access to cellphones among patients on ART and their willingness to receive text message medication reminders. The result shows that the access to cellphones among patients on ART at University of Gondar Hospital is high, with three quarters of patients having access 76.2 % (95 % CI: 71.6–80.0 %). The study also shows that half of the patients 51 % (95 % CI: 45.3–56.3 %) are willing to receive text message medication reminders from their ART clinic.

Age, educational status, use of internet and not disclosing HIV status to anyone other than their healthcare provider are among the notable factors associated with the willingness of patients to receive text message medication reminders.

Accessibility of patients to cellphone in this study (76.2 %) is slightly lower than similar studies from South Africa 81 % [20], China 88.4 % [25], Vietnam 84 % [30], and the United States 92.3 % [27]. This disparity might be due to the difference in information and communication technology (ICT) infrastructure, ICT development index (IDI) and socioeconomic status among the countries crating the digital divide among countries [15]. According to these results the ownership of cellphones is prevalent among patients on ART, therefore cellphone based interventions to improve ART adherence should be tried and explored further.

The access rate in this study is much higher than the Ethiopian general population access to cellphones that was reported to be only 17 % [17]. It is also higher than a study from Uganda 64 % [31]. Those differences might be due to the study setting which was a major town in Ethiopia. Here most of the inhabitants obviously have better access to telecommunication services. Because of this, the findings of this study might not be generalizable to other areas of the country, especially in the rural communities. But, cellphone ownership among patients on ART in this study is consistent with similar studies from Peru 77 % [32] and North Carolina, USA 76.5 % [33]. This similarity could be due to the rapid growth cellphone ownership in the towns of Ethiopia.

Almost all patients in this study (95.9 %) are willing to be contacted by the ART clinic via cellphone using either voice call and/or text. Nearly three quarter (70 %) prefer to be contacted verbally and only half of the respondents (50.9 %) are willing to receive text message ART medication reminders. Other studies show different numbers about the willingness to receive text message ART medication reminders. For example it is higher in South Africa 96 % [20], Peru 81 % [32] and China 68.9 % [25] but it is lower in North Carolina, USA 33 % [33]. This discrepancy can be explained by the difference in the educational status of patients. Our analysis indicates that patients who have achieved secondary or above education are more likely to be willing to receive text message medication reminders. Other studies also showed that literacy is a major barrier to the use of text message medication reminders [32, 34]. Patients who are not able to read and write tend to prefer contact from healthcare providers using only voice call. Therefore it is very important to also offer an optional voice call or voice message medication reminder intervention strategy for those who are unable to use text messages.

It could also be assumed that the patient provider relationship affects the willingness of patients to receive text message medication reminders as a study from Peru [35] suggested. However, our study findings do not show significant associations between the patient provider relationship factors and the willingness to receive text message medication reminders.

From those who are willing to receive text message ART medication reminders, 85.1 % are willing to pay for this service on the current tariff. This high proportion might be due to a better economic status of those who are able to use text messages. If patients on ART can afford to pay for text message medication reminders a fee based system can be designed to support their adherence. Additionally cost sharing mechanisms for text message medication reminders could also help to achieve better adherence levels. However, the fee acceptability and the cost effectiveness of cellphone text message based interventions need to be further investigated.

This study identified numerous factors to be significantly associated with the willingness to receive text message reminders. Patients who are from the younger age group and/or have secondary or higher education are more likely to be willing. This result is consistent with a study from China [25]. However, contrary to this finding a study from North Carolina, USA reported that lower educational attainment was significantly associated with the willingness to receive text message medication reminders [33]. The result suggests that implementing text message medication reminders is particularly feasible in the younger age group.

This study shows that travel time, missing health care appointments and ethnicity are not significantly associated with willingness; again contradictory to the finding of the study from North Carolina [33]. The difference might be due to the difference in socioeconomic status and health service accessibility among the study populations.

Moreover, patients who access the internet by using their cellphone were almost four times more likely to be willing to receive text message medication reminders. It was shown that patients who use internet on their cellphone usually have improved health information access [36] and awareness about the importance of text message medication reminders.

An interesting finding is that cellphone usage privacy variables like locking cellphone with password, perceived text message confidentiality, sharing cellphone with others, storing cellphone in a place where others could see and access were not significantly associated with the willingness to receive text message medication reminders. This is due to the high level of HIV status disclosure to family members and the high extent of sharing mobile phones with families. Those who disclose their HIV status could also share their cellphone and patients trust that their family members keep confidentiality of their HIV related information. This could lead patients to be willing to receive text message medication reminders. Thus, we assume that cellphone usage privacy would not be a major concern for implementing text based medication reminders. However, it has to be further explored whether this holds true or whether patients are just not aware of privacy concerns.

### Limitations of the study

The main limitation of this study is the patient population. Because the study is an institution based cross sectional survey, only respondents who came to the ART clinic for meeting their schedule were interviewed. Moreover, the study was done in a hospital based in a major town which could have inflated the accessibility of patients to cellphones and their willingness to receive text message medication reminders. A different result could have come from a large scale population based study. The survey was also interviewer administered and even if we used neutral interviewers, there might be an interviewer and social desirability bias that could have made more participants to respond as being willing. These limitations have to be taken into account when generalizing the results.

## Conclusion

A large proportion of patients on ART at the University of Gondar Hospital have a cellphone. The findings of this study show that the willingness to use cellphone as ART medication reminders is high. Age, educational status, use of internet and HIV status disclosure are the most notable factors that are associated with the willingness of patients to receive text message medication reminders.

## Additional file

Additional file 1:
**Questionnaire.** (DOCX 24 kb)

## Abbreviations

AIDS: Acquired immune deficiency syndrome
AOR: Adjusted odds ratio
ART: Anti-retroviral treatment
CI: Confidence interval
HCP: Health care provider
HIV: Human immune deficiency virus
OR: Odds ratio
PLWA: People living with HIV/AIDS
SPSS: Statistical package for social sciences
USD: United States Dollar
WHO: World Health Organization
