# Supplementary material for: Willingness to receive text message medication reminders among patients on antiretroviral treatment in North West Ethiopia: A cross-sectional study
Source: BMC Med Inform Decis Mak. 2015 Aug 13;15:65. doi: 10.1186/s12911-015-0193-z (PMC4535252; doi:10.1186/s12911-015-0193-z)
Supplement: Additional file 1: — Questionnaire. (DOCX 24 kb) [file 12911_2015_193_MOESM1_ESM.docx]

**Additional file 1: Questionnaire**

**Section 1: Socio-demographic characteristics**

| **S.No** | **Question** | **Response** | **Skip** |
| --- | --- | --- | --- |
| 101 | Record sex of respondent | 1. Male 2. Female |  |
| 102 | How old are you? | ---------years |  |
| 103 | What is your religion? | 1. Orthodox 2. Muslim 3. Protestant 4. Jewish 5. Other specify |  |
| 104 | Ethnicity | 1. Amhara 2. Tigre 3. Oromo 4. Other specify------------- |  |
| 105 | Marital status | 1. Single 2. Married 3. Separated 4. Divorced 5. Widow/erd 6. Other specify……………. |  |
| 106 | Educational status | 1. Illiterate 2. Read and write 3. Primary 4. Secondary 5. Higher |  |
| 107 | Occupation | 1. Jobless 2. Daily Labourer 3. Government employee 4. Merchant 5. Farmer 6. Driver 7. House wife 8. Student 9. Others Specify………… |  |
| 108 | Average monthly income | 1. < 500 Birr 2. 500-999 Birr 3. >1499 Birr 4. I don’t know |  |
| 109 | Whom do you live with? | 1. Live alone 2. With my spouse 3. With parents 4. Unstable 5. Don’t need to specify |  |
| 110 | Time since HIV diagnosis | 1. 0 to 6 months 2. 7 to 12 months 3. > 12 months |  |
| 111 | Time since started ART | 1. 0 to 6 months 2. 7 to 12 months 3. > 12 months |  |

Section 2: Environmental factors

| 201 | Is there Television in your house? | 1. Yes 2. No |  |
| --- | --- | --- | --- |
| 202 | Is there radio in your house? | 1. Yes 2. No |  |
| 203 | How do you travel to come here for the ART service? | 1. On foot 2. By car 3. By animal 4. Other specify………….. |  |
| 204 | How much time does it take you to come here in your routine way of transportation? | 1. Less than 1 hour 2. More than 1 Hour |  |
| 205 | Is there electricity in your house? | 1. Yes 2. No |  |

Section 3: Patient Provider relationship

| 301 | Are you satisfied with the clinicians service | 1. Yes 2. No |  |
| --- | --- | --- | --- |
| 302 | Do you have open communication with HCP treating you? | 1. Yes 2. No |  |
| 303 | How often do you visit your doctor | 1. every month 2. every 2 month 3. every 3 month 4. Variable |  |
| 304 | Do you obtain the education or  Assistant you need during your visits? | 1. Yes 2. No 3. Not sure |  |
| 305 | Do you have access to reliable pharmacy any time you want? | 1. Yes 2. No 3. Not sure |  |
| 306 | Are you satisfied by the changes/ improvements you obtain for your treatment? | 1. Yes 2. No 3. Not sure |  |
| 307 | Are you satisfied in the scheduling appointments and confidentiality of the treatment unit? | 1. Yes 2. No 3. Not sure |  |
| 308 | Have you ever missed your healthcare appointments? | 1. Yes 2. No |  |
| 309 | If your answer for the above question is yes, What was the reason to miss your appointment? | 1. I forgot it 2. I was sick and unable to come myself 3. I didn’t get permission from my employers 4. Other specify……… |  |
| 310 | Do you miss taking your medication? | 1. Yes 2. No |  |
| 311 | How often do you miss your medications | 1. I miss my medications everyday 2. At least once in a week 3. More than once in a week 4. I never miss taking medications 5. Other specify…. |  |
| 312 | What is the reason to miss taking your medications? More than one answer possible | 1. I was too busy with other things or simply forgot. 2. I was away from home. 3. There was a change in my daily routine. 4. I felt asleep. 5. I felt depressed or overwhelmed. 6. I had problem taking medication at specific times. 7. I felt sick or ill at that time 8. I ran out of medication. 9. I had too many pills to take. 10. I felt the drug is too toxic/ harmful and want to avoid side effects. 11. I did not want other to notice me I am taking medicine. 12. Taking the drugs is a reminder of my HIV. 13. I was confused about the dosage directions at that time. 14. I did not think the drug is doing anything to improve my health. 15. People told me that the medicine is not good. |  |

Section 4: Psychological factors

| 401 | Do you have a sense of care, safety, security of support from your family, co-workers, fewer do or other people in your common? Yes No | 1. Yes 2. No 3. Not sure |  |
| --- | --- | --- | --- |
| 402 | What kind of support or care you obtain from the above people? | 1. Material / practical  2. Information / advice  3. Other specify…………… |  |
| 403 | Are you satisfied with their help? | 1. Yes 2. No |  |
| 404 | Are you esteemed or valued for you skills or abilities by other? | 1. Yes 2. No |  |
| 405 | Are you satisfied with the way people hold you in esteem or value for your skills or abilities? | 1. Yes 2. No |  |
| 406 | Are you fully convinced that you are infected I HIV and needs ARV | 1. Yes 2. No |  |
| 407 | Do you have any doubts about HIV/ ARV, HCP? | 1. None 2. Some 3. Many |  |
| 408 | Do you think this treatment benefits you? | 1. Yes 2. No |  |
| 409 | Do you feel confident about your ability to lake the medication accordingly to the regimen of restrictions or do you have some duet or difficulties? | 1. Yes 2. No |  |

Section 5: Behavioral factors

| 501 | Do you feel comfortable when you take ART in front of others? | 1. Yes 2. No |  |
| --- | --- | --- | --- |
| 502 | Do you use any reminder mechanisms? | 1. Yes 2. No | If No>>>go to Q 504 |
| 503 | If your answer for the above question is yes, What type of reminding mechanism do you use? | 1. Pillbox 2. Written schedule 3. Watch bell 4. Mobile phone 5. Other specify |  |
| 504 | Do you disclose your HIV status? | 1. Yes 2. No | If No>>>>go to Q 506 |
| 505 | If your answer for Q504 is yes, for whom did you disclose your HIV status | 1. Spouse only 2. Spouse + other family members 3. Other relatives 4. Friend only 5. No one |  |
| 506 | Do you take any addicting substances? | 1. Yes 2. No | If No>>>>go to Q 601 |
| 507 | If your answer for Q506 is yes, what kind of substances do you take? | 1. Alcohol 2. Kchat 3. Cigarette 4. Other specify |  |

Section 6: Pattern of cell phone use

| 601 | Do you have mobile phone? | 1. Yes 2. No | If your answer is yes >>>>go to 602, If no stop |
| --- | --- | --- | --- |
| 602 | Do you use this cell phone as your medication reminder | 1. Yes 2. No |  |
| 603 | What is your preferred way of communication in your cell phone? | 1. Verbal 2. Text 3. Email |  |
| 604 | How often do you have your cell phone with you? | 1. Always 2. Sometimes 3. Seldom 4. Never |  |
| 605 | Have you had your cell phone lost, damaged or theft in the past? | 1. Yes 2. No |  |
| 606 | Do you have any other phone number? | 1. Yes 2. No |  |
| 607 | Switch off cell phone during day | 1. Yes 2. No |  |
| 608 | There is sometimes a time or place where no calls are taken | 1. Yes 2. No |  |
| 609 | Are there times that you don’t answer unknown calls? | 1. Yes 2. No |  |
| 610 | Do you use phone pass words? | 1. Yes 2. No |  |
| 611 | Do you put your cell phone in a place where others could use and access? | 1. Yes 2. No |  |
| 612 | Do you share your cell phone with other person? | 1. Yes 2. No |  |
| 613 | Can you read/send text message using your mobile? | 1. Yes 2. No | If No>>>>go to Q618 |
| 614 | If your answer for the above question is yes, do you delete text message without reading it? | 1. Yes 2. No |  |
| 615 | How likely is that a text message received on your phone to be seen by others? | 1. Very likely 2. Somewhat likely 3. Somewhat unlikely 4. Very unlikely |  |
| 616 | Do you use internet on your phone? | 1. Yes 2. No | If “Yes” >>>>go to Q617 |
| 617 | If your answer for Q616 is yes, what is the website page that you most frequently visit? | 1. Social network pages like Face book 2. Email 3. Google 4. Others specify |  |
| 619 | Are you willing to be contacted by your mobile telephone from your health service provider to remind your medications? | 1. Yes I am willing 2. No I don’t like to be contacted |  |
| 620 | How do you want to be reminded? | 1. Mobile phone calls 2. Text messages 3. Mobile phone pager 4. Both are helpful for me |  |
| 621 | Do you think mobile two way SMS could be helpful in your adherence to ART? | 1. Yes 2. No | If “no” >>>>go to Q622 |
| 622 | If your answer for the above question is no, what do you think is bad to receive text message reminder? | 1. It ruins my privacy 2. Text message from one’s healthcare provider would be annoying 3. Other specify |  |
| 623 | Will you pay for text message service you send to your clinic to remind your medication and appointments according to the current telecommunication tariffs? | 1. Yes 2. No |  |
| 624 | If we were going to develop an application for people living with HIV in our hospital using cell phones – what sort of things would you like to see? More than one answer is possible | 1. Automatic medication reminders 2. Automatic appointment reminders 3. Health advices/tips 4. Other specify |  |

Thank you for your cooperation.
